# Anethole induces anti-oral cancer activity by triggering apoptosis, autophagy and oxidative stress and by modulation of multiple signaling pathways

**DOI:** 10.1038/s41598-021-92456-w

**Published:** 2021-06-22

**Authors:** Camille Contant, Mahmoud Rouabhia, Lionel Loubaki, Fatiha Chandad, Abdelhabib Semlali

**Affiliations:** 1 Groupe de recherche en écologie buccale, Faculté de médecine dentaire, Université Laval, Québec, QC G1V 0A6 Canada; 2 Department of Biochemistry, Microbiology and Bioinformatics, Laval University, Québec, QC Canada

**Keywords:** Cancer, Molecular biology, Plant sciences, Medical research

## Abstract

Oral cancer is one of the major public health problems. The aim of this study was to evaluate the effects of anethole, 1-methoxy-4-[(E)-1-propenyl]-benzene, on growth and apoptosis of oral tumor cells, and to identify the signaling pathways involved in its interaction with these cancer cells. Cancer gingival cells (Ca9-22) were treated with different concentrations of anethole. Cell proliferation and cytotoxic effects were measured by MTT and LDH assays. Cell death, autophagy and oxidative stress markers were assessed by flow cytometry while cell migration was determined by a healing capacity assay. The effect of anethole on apoptotic and pro-carcinogenic signaling pathways proteins was assessed by immunoblotting. Our results showed that anethole selectively and in a dose-dependent manner decreases the cell proliferation rate, and conversely induces toxicity and apoptosis in oral cancer cells. This killing effect was mediated mainly through NF-κB, MAPKinases, Wnt, caspase 3, 9 and PARP1 pathways. Anethole showed an ability to induce autophagy, decrease reactive oxygen species (ROS) production and increased intracellular glutathione (GSH) activity. Finally, anethole treatment inhibits the expression of oncogenes (cyclin D1) and up-regulated cyclin-dependent kinase inhibitor (p21^WAF1^), increases the expression of *p53* gene, but inhibits the epithelial-mesenchymal transition markers. These results indicate that anethole could be a potential molecule for the therapy of oral cancer.

## Introduction

Oral cancer is amongst the world’s highest public health threat issues^1^. Ranking in 11th position as most common cancer in the world, it can cause more than 145,000 deaths a year worldwide, with higher prevalence in men compared to women^2^. It varies depending on socioeconomic and environmental conditions. Multiple factors such as alcohol, tobacco, malnutrition, viral infection and genetic aspect can play a major role in the etiology of the disease^2^. Oral cancer is a squamous carcinoma frequently identified on lips, gums, tonsils, tongue, salivary glands, back of the throat, floor of the mouth and palate, inside of cheeks and oropharynx^3^. A high level (52.7%) of oral cancer is diagnosed with stage IV of cancer development^4^. Conventional treatments for oral cancer rely heavily on surgery with or without assistance of chemo- and radiotherapy care^5^. Although they are efficient, both have significant adverse effects limiting their use^6^. Those treatments can therefore be more aggressive causing numerous unwanted side effects and can have a negative impact on non-cancer cells in mucosa tissue^7,8^. To lower these adverse effects, plant products may be used as complementary treatments in conjunction with reduced exposure to chemo- and radiotherapy. For decades, in countries such as China, India, and Egypt, traditional medicine has been making use of flavored plants as home-based treatments of many diseases including cancer^9^. Herbal ingredients were shown to inhibit proliferation of cancer cells and to reduce side effects caused by chemo- and radiotherapy treatments^10^. Traditional medicine could therefore be an effective combination or alternative treatment for cancer that may be efficient and more specific than compounds like cisplatin used in chemotherapy^11^. Indeed, many bioactive compounds derived from scented plants, like anethole, 1-methoxy-4-[(E)-1-propenyl]-benzene, have been reported to have anti-cancer properties on MCF-7 and PC-3 cell lines^12,13^. Indeed, it has been demonstrated that anethole inhibits cell growth, induces apoptosis and cytotoxicity towards cancer cell lines. However, no study has yet investigated the effects of anethole on oral squamous carcinoma and on normal cells of mucosa.

Cancer initiation and development involve signaling pathways such as the activation of caspases, MAP kinases, and Wnt^14,15^. Anticancer treatment can include one or all these signaling pathways. It was reported that identical mutations downstream of MEK/ERK proteins of EGFR pathway, particularly in KRAS and BRAF, were highly frequent in cancers^16^. Therefore, the objective of this study was to investigate the effects of anethole on the proliferation, apoptosis, autophagy and oxidative stress of Ca9-22 cells. We also assessed the effects of anethole on cell migration and modulation of pro-oncogenic (cyclin D1), apoptotic proteins, epithelial-mesenchymal transition (E-cadherin, vimentin), MAPK (ERK1/2, p38, Jnk), Wnt (β-catenin) and NF-κB signaling pathways. The different results could help understanding the anti-cancer activities of anethole which may lead to an alternative or complementary anti-oral cancer therapy.

## Materials and methods

### Cells and cell culture conditions

The gingival epithelial cancer cell line (Ca9-22) was obtained from RIKEN BioResource Research Center, Tsukuba, Japan. Ca9-22 cells were cultured in RPMI-1640 medium (Thermo Fisher Scientific, Burlington, ON, Canada), supplemented with l-glutamine, 5% fetal bovine serum (FBS) (Gibco; Thermo Fisher Scientific, Burlington, ON, Canada) and antibiotics (Sigma-Aldrich, Oakville, Ontario, Canada). Normal primary human gingival epithelial cells (GEC) and normal fibroblasts (GF) were isolated from normal human gingival tissues of healthy donors (18 to 30 years old) following an informed consent signature and we confirm that all protocols are carried out in accordance with relevant guidelines and regulations. Tissue collections were approved by the Ethics Committee of Université Laval. The gingival epithelial cells were cultured in Dulbecco’s modified Eagle’s/Ham’s F12 (3∶1; DMEH) medium (Gibco; Thermo Fisher Scientific) supplemented with growth factors and 10% fetal calf serum^17^. The gingival fibroblasts were maintained in Dulbecco’s modified Eagle’s (DME) medium (Gibco; Thermo Fisher Scientific) containing 10% fetal calf serum (Invitrogen Canada Inc., Burlington, ON, Canada)^18^. Cell cultures were performed at 37 °C in humidified incubator with 5%; CO_2_ atmosphere conditions. Normal human gingival epithelial cells were used at passages 3 to 4, and normal human gingival fibroblasts were at passages 4 to 5 in our experiments.

### Drugs

Anethole was purchased from Sigma-Aldrich (Oakville, ON, Canada). It was diluted in methanol to reach a stock solution of 3 mM and used at various concentrations (0, 0.3, 3 and 30 μM).

### Effect of anethole on the morphology and LDH activity of normal and cancer gingival cells

Cells were seeded at 10^5^/well in 6-well cell culture plates, and exposed to various concentrations (0, 0.3, 3 and 30 μM) of anethole for 24 h. The cell morphology was evaluated by means of an inverted optical microscope (Eclipse TS100, Nikon, Montreal, QC, Canada) and photographed with a digital camera. Each experiment was repeated three times. The lactate dehydrogenase (LDH) activity was assessed in culture supernatants collected after cell exposure to anethole for 24 h. The LDH activity was measured using LDH (Sigma-Aldrich, Oakville, ON, Canada) as described previously^19^. Each experiment was repeated seven times.

### Effect of anethole on cell proliferation

Normal and cancer gingival cells (3 × 10^5^ cells/well) were seeded in 6-well plates and cultured for 24 h. Cells were then exposed or not to various concentrations of anethole for 24 h, then sued to assess the cell growth by means of MTT colorimetric assay (Sigma-Aldrich) as described by Semlali et al.^20^ Briefly, each culture was supplemented with 10% of MTT (5 mg/mL) and incubated for 3 h at 37 °C. Stained cells were then lysed using 1 mL of isopropanol-HCl 0.05 M solution with agitation for 15 min. Then, 4 × 200 µL of lysis solution were transferred to a 96-well plate and the absorbance was measured at the wavelength of 550 nm using an iMark microplate reader (Bio-Rad, Mississauga, ON, Canada). Cell proliferation levels were determined by means of the following formula: % of cell viability = [(OD _550 nm_ (treated cells) − OD (Blank))/(OD (control cell) − OD (Blank))] × 100, n = 7. With another set of experiments, anethole treated and non-treated gingival cells were detached by means of 0.05% trypsin: 0.1% EDTA solution (Sigma-Aldrich, Oakville, ON, Canada) and resuspended in fresh culture medium. The level of living cells in each culture was determined by means of the trypan blue exclusion assay, as described previously^21^.

### Colony formation assay

Ca9-22 cells were seeded on six-well plates at 1.10^3^ cells per well during overnight for adhesion and treated with various anethole concentrations (0–0.3–3–10 and 30 µM) for two weeks. After time of treatment, the survival clones were fixed by 4% of formaldehyde for 20 min, washed twice by PBS before staining with 0.5% crystal violet for 30 min, and photographed with a digital camera.

### Cell cycle distribution

The percentage of cell distribution for each phase of cell cycle was evaluated by using the fluorescent DNA dye 7-aminoactinomycin D (7-AAD) (Sigma, USA). Briefly, oral cancer cells were treated with different concentrations of anethole (0 to 30 µM) for 24 h. They were then incubated in the presence of 7-AAD at 1 μg/mL for 30 min at 37 °C. The fluorescence intensities of DNA were detected with “LSRII” or “CantoII” (BD Biosciences). Data were analyzed by FACSDiva software v. 6.1.3 and experiments were done in triplicates, n = 4.

### Cell apoptosis assay by annexin V-FITC and propidium iodide

Ca9-22 cell cultures were treated or not with different concentrations (0 to 30 μM) of anethole and incubated for 24 and 48 h in a 5% CO_2_ humid atmosphere at 37 °C. The cells were then detached with a 0.05% trypsin:0.1% EDTA solution and used to assess apoptosis using Annexin V-FITC/propidium iodide kit (BD Bioscience, Mississauga, ON, Canada). Following staining, cell suspensions were analyzed by flow cytometry (BD Accuri™ C6; BD Bioscience, Mississauga, ON, Canada). The experiment was repeated three times.

### Effect of anethole on cancer gingival cell migration

Ca9-22 cells were seeded in 6-well plates and cultured to 100% confluence. A cross-shaped scratch was created on each confluent monolayer with a sterile 200 μL pipette tip. Culture medium was then refreshed, and cells were incubated with or without 3 μM of anethole at 37 °C in a humid atmosphere containing 5% CO_2_. Digital photographs of each lesion were taken with an inverted optical microscope at different times (0, 6, 12 and 24 h) post injury. Wound closure was analyzed using an image processing program to measure the distance, at 3 separate sites, between opposite edges at each time point (0, 6, 12 and 24 h). Stimulated and non-stimulated cell cultures were compared with respect to their percentage of closure. The experiment was repeated four times.

### Effect of anethole on cell autophagy

To determine the *in vitro* effect of anethole on Ca9-22 cells autophagy, we used ICT’s Autophagy assay (Red from ImmunoChemistry Technologies, Burlington, ON, Canada). After treatment or not with anethole for 24 h, the Ca9-22 cells were detached and cultured with the autophagy solution (1:50), incubated in the dark for 1 h, washed three times with the cellular assay buffer, and resuspended in 0.5 mL of the same buffer. Stained cells were analyzed by flow cytometry using a green/yellow laser. The Autophagy Probe, Red excites at 590 nm and emits at 620 nm wavelength. The experiment was repeated three times.

### Determination of ROS and GSH levels

Oxidative stress was assessed by flow cytometry using two markers, reactive oxygen species (ROS) and intracellular glutathione (GSH) (ImmunoChemistry Technologies, Burlington, ON, Canada). Firstly, cells were cultivated for 24 h with or without anethole. Second, total ROS Green was reconstituted with DMSO, diluted in the buffer, added to each sample (10^6^ cells/mL), and incubated in the dark for 1 h at 37 °C. The key reagent can then be detected by flow cytometry depending on presence of ROS. Lastly, a decrease of intracellular GSH, a molecule with reducing power playing a key role against free radicals and toxins, is an indicator of the induction of apoptosis in cells stressed by oxidation. To evaluate levels of intracellular GSH, ThioBright™ Green reagent was reconstituted in DMSO, added to the cells suspension (1:200), and incubated in the dark for 30 min. After washing with PBS twice, the intensity of fluorescence was analyzed by BD Accuri C6 Flow Cytometry system (BD Bioscience) and the percentage of positive cells was calculated. Each experiment was repeated three times.

### Western blotting

Gingival cancer cells were exposed to anethole (10 μM) for 24 h, then total proteins were extracted from each condition, by adding 70 μL of the lysis buffer (25 mM Tris-HCl, pH 8.0, 0.15 M NaCl, 1 mM EDTA, 10% glycerol, 0.1% SDS, 0.05% sodium deoxycholate and 1% Triton X-100) to the cell pellet. This buffer was supplemented with 1:100 protease and phosphatase inhibitors (Sigma-Aldrich, Oakville, Ontario, Canada). The solution was kept at 4 °C for 1 h with shaking before being centrifuged at 13,000 rpm for 10 min to recover the supernatant. Protein concentration was determined by the Bradford assay using Coomassie Brillant Blue G-250 Dye (Bio-Rad, Mississauga, ON, Canada). Extracted proteins were subjected to Western blot analyses. Proteins (20 μg to 50 μg) were denatured at 95 °C for 5 min by adding a reducing buffer (61.5 mM Tris, 2% SDS, 10% glycerol and 100 mM dithiothreitol). Then, these were separated by SDS-PAGE with a Bio-Rad electrophoresis system. Briefly, 8% to 15% of acrylamide SDS-PAGE were used depending on protein molecular weight. After protein migration and transfer onto polyvinylidene difluoride membrane (PVDF) (Cytiva, Vancouver, BC, Canada). The membrane was incubated in a blocking buffer (5% non-fat milk in a solution of TBS 1x) with shaking for 1 h at room temperature followed by overnight incubation with appropriate primary antibodies: Cyclin D1 (sc-8396), p21 (sc-6246), p53 (sc-263), Bax (sc-7480), Bcl-2 (sc-509), Procaspase 3 (sc-56046), Procaspase 9 (sc-17784), total PARP-1 (sc-8007), NF-κB (sc-8008) and β-Catenin (sc-59737) were purchased from Santa Cruz Biotechnology (Santa Cruz, CA, USA), E-Cadherin (8834), pERK1/2 (4370), ERK1/2 (4695), pp38 (4631), p38 (9212), pJNK (4668P), JNK (9252S), Cleaved caspase-3 (9664S), Cleaved caspase-9 (20750S), Cleaved PARP-1 (5625S), Cytochrome C (11940S), LC3B (2775) and SQSTM1/p62 (39749) were from Cell Signaling Technology (Danvers, MA, USA), Vimentin (Ab8978) from Abcam (Cambridge, MA, USA) and β-actin (A5441) was purchased from Sigma-Aldrich (Oakville, ON, Canada). The secondary goat anti-mouse (554002) and anti-rabbit (554021) were from BD Pharmingen (Mississauga, ON, Canada). The membrane was washed 4 ×10 min with TBS 1x + 0.05% Tween 20 solution. The conjugated secondary antibodies were then applied for 1 h and the membrane was washed as previously. The detection was carried out using the Clarity Western ECL Substrate (Bio-Rad, Mississauga, ON, Canada). The visualization was with VersaDoc MP 5000 system (Bio-Rad, Mississauga, ON, Canada). Each experiment was repeated three times.

### MMPs and TIMPs secretion

Supernatants that are being collected from each culture condition were subjected to an xMAP technology (Luminex, Austin, TX, USA) for multiplexed quantification of human cytokines, chemokines and growth factors by Eve Technologies Corp (Calgary, Alberta). Thirteen markers were simultaneously quantified in samples using the Human MMP Magnetic Luminex Performance Assay 9-Plex Panel and the Human TIMP Magnetic Luminex Performance Assay 4-plex Fixed Panel (R&D Systems, Minneapolis, MN, USA) according to the manufacturer’s protocol. The 9-plex consisted of MMP-1, MMP-2, MMP-3, MMP-7, MMP-8, MMP-9, MMP-10, MMP-12 and MMP-13 and as for the 4-Plex, of TIMP-1, TIMP-2, TIMP-3 and TIMP-4. The assay sensitivities of these markers range from 0.5 to 86.0 pg/mL for the 13-plex. Values for individual analyte are available in R&D Systems protocols. Each experiment was repeated three times.

### Statistical analysis

Each experiment was performed at least three times, with experimental values expressed as means ± SD. The statistical significance of differences between values for the control (absence of anethole) and the test (presence of desired concentration of it) was determined by a percentage. The statistical significance of differences between the values was determined by using a one-way ANOVA. *p*-value < 0.05 was defined as significant.

## Results

### Anethole has selective cytotoxic effect on oral cancer cells

**Figure 1:**
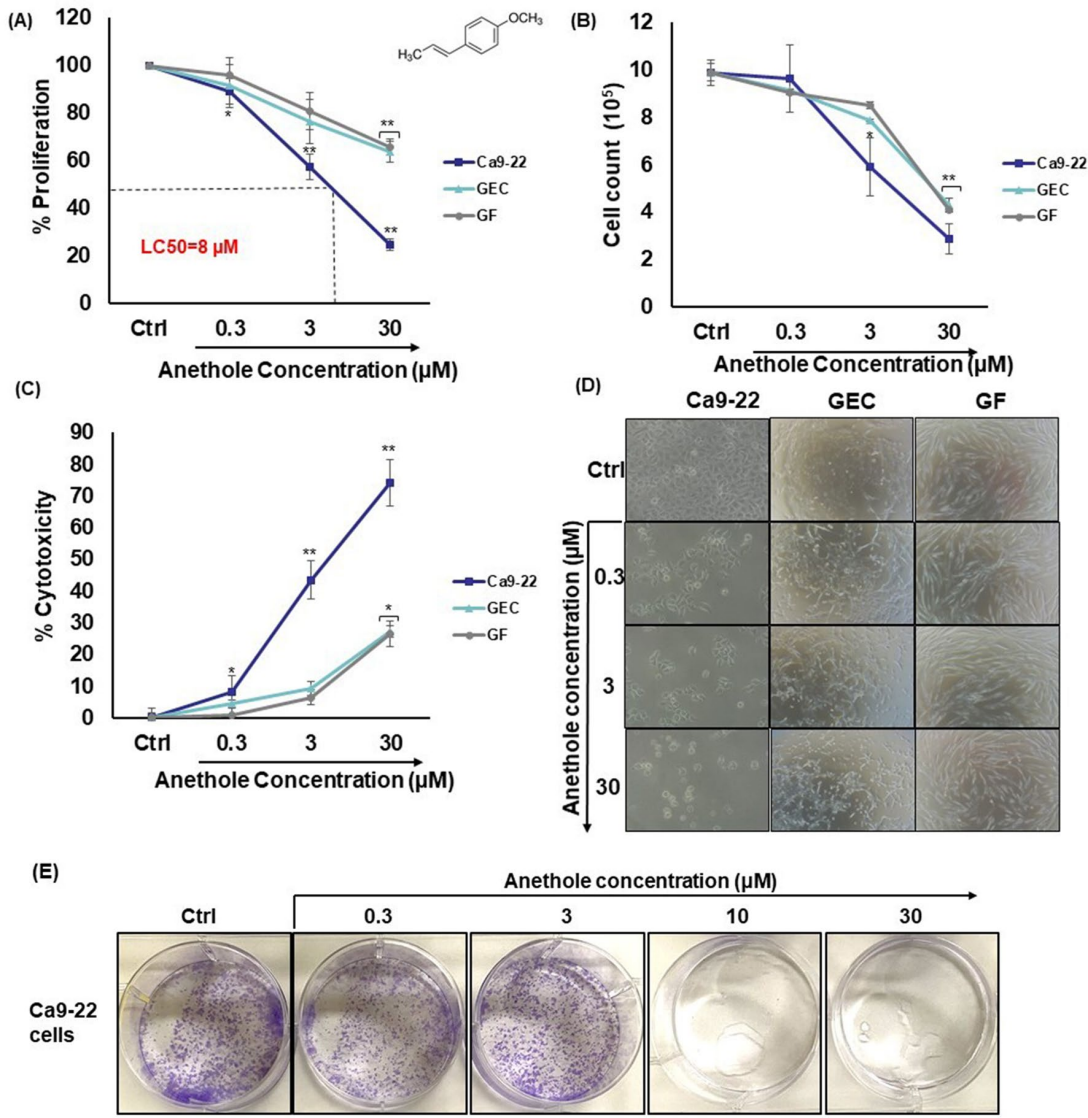
Anethole selectively inhibits the growth of oral cancer cells. (**A**) Cell growth was assessed by MTT assay. Cells were exposed to different concentrations of anethole for 24 h. Results are reported with means (% of proliferation) ± SD and the control represents 100% of proliferation, on Ca9-22 cells (n = 10), GEC (n = 3) and GF (n = 4). (**B**) Cytotoxicity was assessed by LDH assay using supernatant. Cells were exposed with anethole, ranging from 0.3 to 30 μM for 24 h. Results are reported with means (% cytotoxicity) ± SD and Triton was used as positive control. Significance was obtained when comparing the control to anethole treated cells. *p < 0.05 or **p < 0.005. (**C**) Nuclear staining. Cells were first cultured in the presence or absence of anethole for 24 h. Attached cells were then stained with Hoechst (n = 3). (**D**) The morphology of gingival epidermoid carcinoma cells (Ca9-22), healthy epithelial cells (GEC) and normal gingival fibroblasts (GF) was observed after 24 h of stimulation with different concentrations of anethole, ranging from 0.3 to 30 μM. (n = 3). (**E**) Cell viability of oral cancer cells with anethole treatments was determined by clonogenic assay at 2 weeks (n = 3).

We have investigated the cytotoxic effect of anethole on oral cancer cells (Ca9-22), non-tumorigenic primary gingival epithelial cells and fibroblasts (GEC and GF) using four types of experiment. At first, growth and cytotoxic effect were measured by MTT and cell count assays (Fig. 1A,B). While GEC and GF cells exhibited great resistance to anethole, with an LC_50_ (the concentration that leads to 50% mortality) greatly exceeded 30 µM for GEC as well as GF, the cancer cell (Ca9-22) line showed clear sensitivity to anethole even at low concentrations. Indeed, the LC_50_ was approximately 8 µM. The proliferation level of Ca9-22 cells decreased from 11.0% ± 5.5% (*p* = 0.04), 42.7% ± 5.2% (*p* = 1 × 10^−5^) to 75.4% ± 2.3% (*p* = 6 × 10^−11^), respectively with anethole concentrations of 0.3, 3 and 30 μM. Using normal gingival epithelial cells (GEC), a slight decrease (36.3% ± 4.3% [*p* = 0.007]) of cell growth was observed with 30 μM of anethole. Similar observation was reached with normal gingival fibroblasts showing 34.2% ± 3.6% (*p* = 0.001) growth decrease with 30 μM of anethole. Cell counting confirms the MTT results. They also correlated with the LDH assay, as shown in Fig. 1C. Indeed, anethole concentration, from 0.3 to 30 μM, induced a dose-dependent high cytotoxicity in Ca9-22 cells, ranging from 8.2% ± 5.1% (*p* = 0.04) to 74.2% ± 7.3% (*p* = 2 × 10^−5^), compared to only 27.3% ± 1.8% (*p* = 0.02) for normal gingival epithelial cells and 26.5% ± 4.0% (*p* = 0.003) for normal fibroblasts. These outcomes indicate that anethole is having differential cytotoxicity against oral cancer cell lines, but it seems less toxic against non-neoplastic gingival epithelial cells and fibroblasts. Microscopic analysis of the morphology is indicating a qualitative change in gingival cancer cells when treated with low concentration (3 µM) of anethole unlike in normal gingival cells (GEC and GF). Anethole is considerably decreasing the adherence of oral cancer cells (Ca9-22), promoting an irregular shape as well as the presence of dose-dependent granules, while it has low morphological damage with normal gingival cells (Fig. 1D). For clonogenic assay (Fig. 1E), cell viability of the Anethole treatment was significantly lower than the untreated cells (control) especially at 10 µM and 30 µM of anethole.

### Anethole affects the cell cycle distribution in oral cancer cells

**Figure 2:**
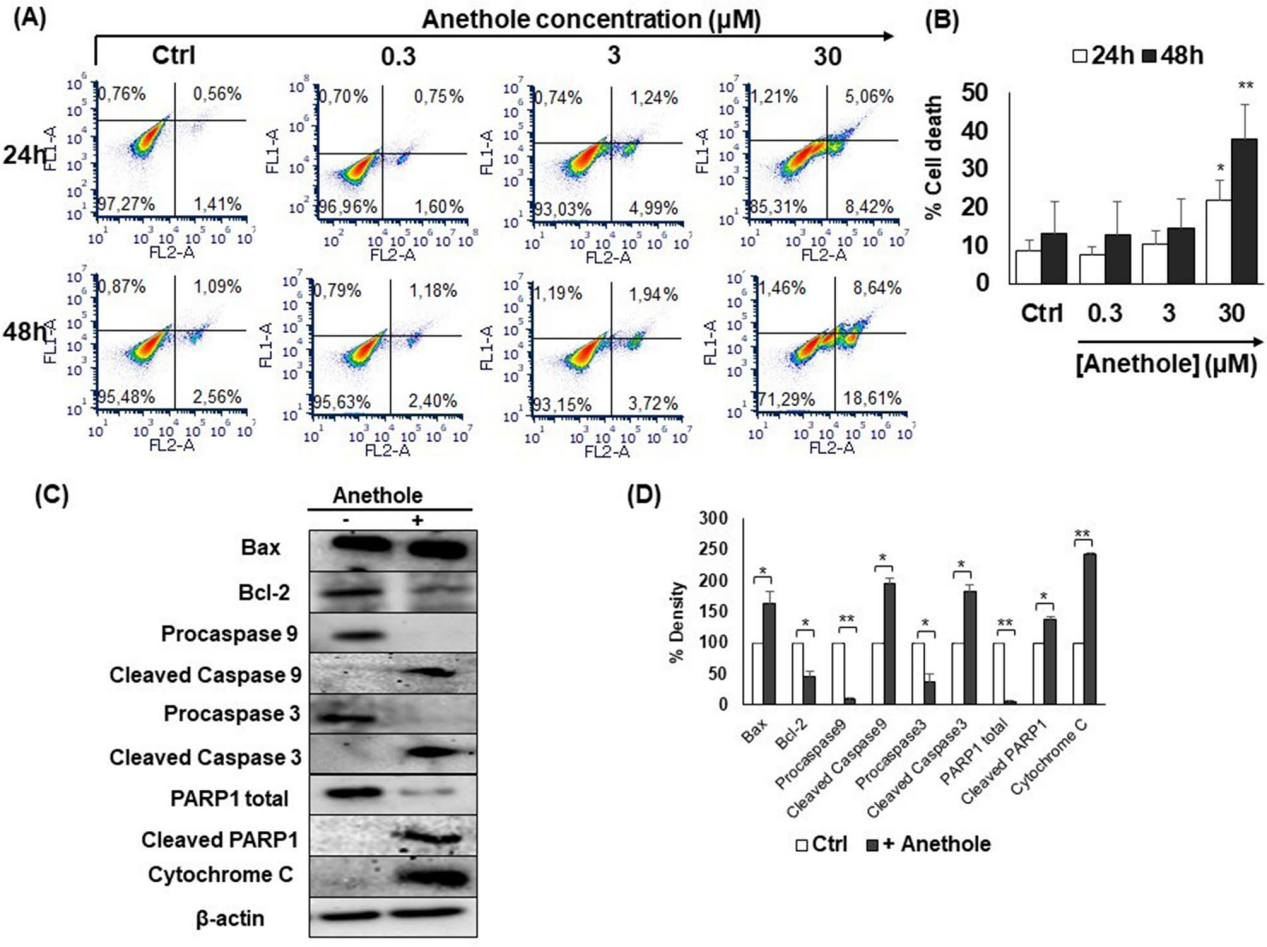
Anethole induces apoptosis of oral cancer cells. (**A**) Mouth epidermoid carcinoma cells (Ca9-22) were exposed to different concentrations of anethole (0.3 μM to 30 μM) for 24 h or 48 h. Apoptosis/necrosis was measured with an annexin V/PI kit and analyzed by flow cytometry (n = 3). Controls was being used to determinate the gates. (**B**) Histogram displays flow cytometry results of Ca9-22 cell apoptosis/necrosis after 24 h and 48 h of anethole exposure. Results are reported with means (% of death) ± SD. (**C**) Western blotting confirmed the involvement of apoptotic pathways and expression after being exposed with anethole 10 μM for 24 h. Proteins were extracted with the Bradford technique before migration on SDS-PAGE (7 to 15%). β-actin was used as control (n = 3). (**D**) Represents a histograms showing fold changes of indicated proteins. 100% was the density of untreated cells.

To study its effect on the cell cycle distribution in Ca9-22 cells, they were treated with various concentrations of anethole from 0 to 30 µM for 24 h. The percentage of cell distribution, in each phase of cell cycle, was evaluated by staining with 1 μg/mL of 7-AAD and by using a flow cytometer, as described in the Materials and Methods section. Figure 2 summarizes the means of 4 experiments, which shows that anethole, at 3 µM and 30 µM, reduced significantly the percentage of Ca9-22 cells in G0/S phase, from 75.9% with the control, to 44.5% with anethole at 3 µM, and 50.9% at 30 µM. In addition, the percentage in the S phase was significantly increasing (*p* < 0.005), from 2.8% with the control, to 11.2% and 6% respectively when the cells were treated at 3 µM and 30 µM. Also, the percentage of Ca9-22 cells at G2/M phase increased with anethole stimulation, from 3.3% with the control, to 12.2% with 3 µM and 5.1% at 30 µM (Fig. 2). These outcomes are suggesting that anethole could induce S and G2/M cell cycle arrest in Ca9-22 cells.

### Anethole triggers apoptosis of oral cancer cells via the mitochondrial pathway

To understand the mechanism by which anethole plays a potential anti-oral cancer role, we have investigated its apoptotic effect on Ca9-22 cells using Annexin V measurement by flow cytometry. Figure 2A shows that anethole at 30 μM induces more than 29% of cells towards apoptosis after 48 h of treatment. As for Fig. 2B, it compares a proposed model of anethole-induced cell death (early and late apoptosis) with non-treated cells after 24 h and 48 h. This data is indicating that anethole induces cytotoxicity through an apoptosis pathway. To determine the apoptotic mechanism activated by anethole, proteins were analyzed by western blot showing an increase of the Bax/Bcl-2 ratio, due to an increase of the Bax (pro-apoptotic) expression, and a decrease of Bcl-2 (anti-apoptotic) proteins. Our results showed that anethole is triggering cell death in oral cancer cells through induction of caspase 3 and PARP-1 active forms (Fig. 2C). Furthermore, anethole is strongly inhibiting the level of total caspase 9 but increasing the expression of caspase 9 and cytochrome C active forms after 24 h of treatment (Fig. 2C,D). The results suggest that anethole activated intrinsic pathways via caspases and PARP1.

### Anethole suppresses migration/invasion abilities of oral cancer cells and mediates the anti-metastatic activity in the latter by suppressing epithelial-to-mesenchymal transition

**Figure 3:**
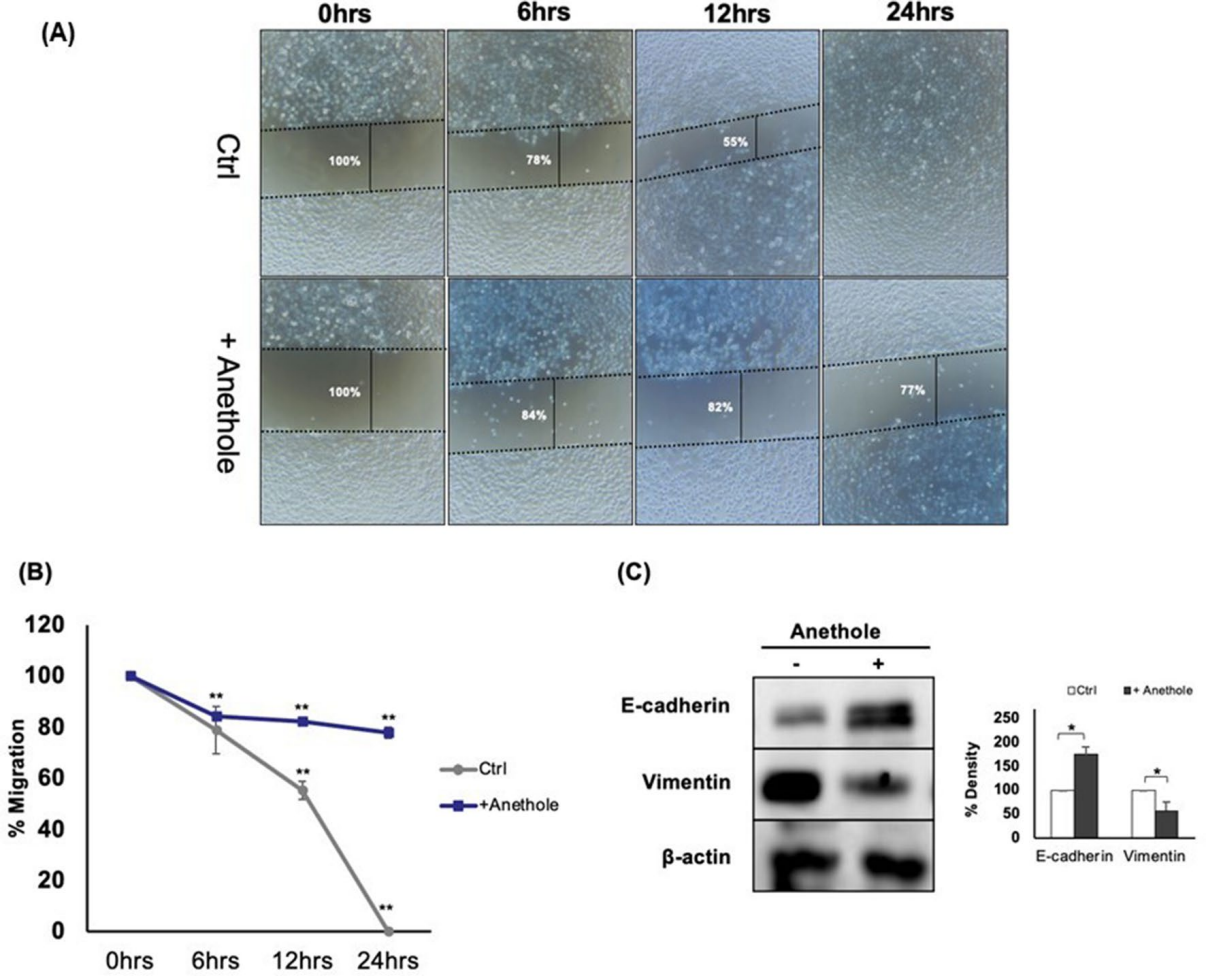
Anethole inhibits the migration of oral cancer cells. (**A**) Mouth epidermoid carcinoma cells (Ca9-22) were cultured up to 100% confluence. Scratches were then made on each monolayer and cells were incubated with or without anethole. Cell migration/wound repair were observed over time (0, 6, 12 and 24 h) and pictures were taken. Wound closure was measure by the distance, at 3 separate sites, between opposite edges of it. Results are reported as the means (%) ± SD (n = 3). (**B**) Graphs displaying the three experiments of a cell lesion assay on Ca9-22 incubated with or without anethole 3 μM. (**C**) Western blotting of E-cadherin and vimentin after being exposed for 24 h, with or without anethole (10 μM) (n = 3).

One of the cancer cell properties is the migration/invasion, thus treatment should contribute inhibiting cancer cell migration. In this study section, we investigate the effect of anethole on Ca9-22 cells migration after scratch. As shown in Fig. 3A,B, anethole is repressing the migration of oral cancer cells. Only 23% of the area was covered with migrating cells following treatment with 3 µM of anethole, while 100% of the scratch area was covered with migrating cells in the untreated control. Cancer metastases are closely associated with induction of epithelial-to-mesenchymal transition (EMT), which is characterized by increased cell motility, loss of cell adhesion, and repression of E-cadherin expression. In the current study, Western blotting showed that anethole at 10 µM is enhancing E-cadherin expression, an EMT epithelial marker, but attenuating that of vimentin, a mesenchymal marker (Fig. 3C). These results imply that anethole inhibits oral cell migration and blocks the epithelial-to-mesenchymal transition in oral cancer cells.

### Anethole promotes autophagy of oral cancer cells by induction of LC3B and p62 expressions

**Figure 4:**
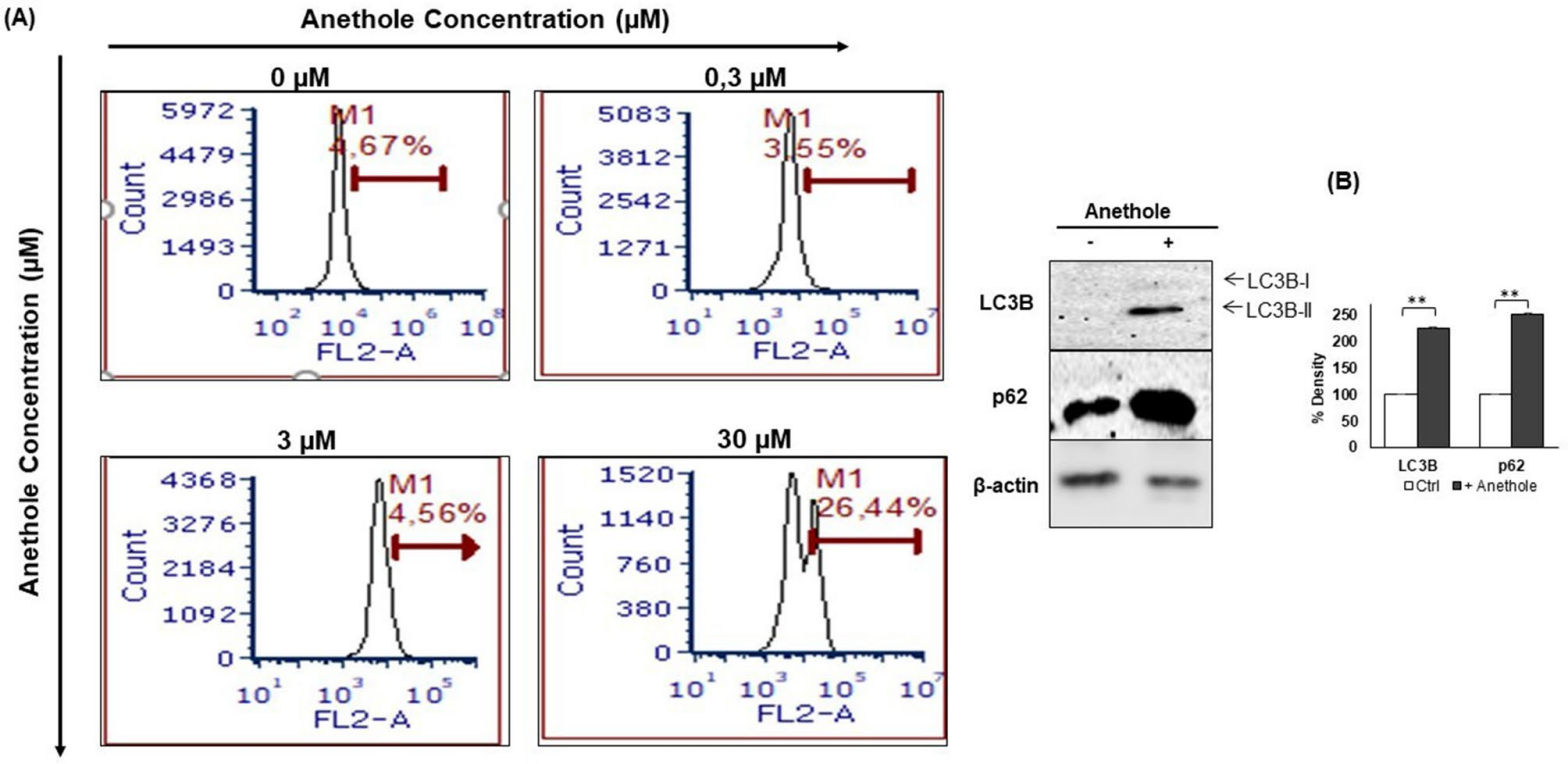
Anethole induces autophagy of oral cancer cells. (**A**) represents the flow cytometry analysis (n = 3). The results were reported with the means (% autophagy) ± SD and are considered significant when *p < 0.05. (**B**) Protein level of LC3B by western blotting (n = 3).

We further investigated whether different concentrations of anethole induced the progression of autophagy. The results (Fig. 4A) suggested that anethole (30 μM) promotes autophagy of cancer cells. Indeed, after 24 h of incubation with or without anethole (0, 0.3, 3 and 30), results showed an increase in autophagy ranging, respectively, from 0.07% ± 0.07% to 4.3% ± 4.3% (*p* = 0.3), 2.0% ± 1.8% (*p* = 0.3) and 39.6% ± 13.3% (*p* = 0.04). Compared to the control, the treatment at 10 μM of anethole significantly induced the overexpression of LC3B and p62 protein levels in the Ca9-22 cells (Fig. 4B).

### Anethole inhibits oxidative stress in oral cancer cells

**Figure 5:**
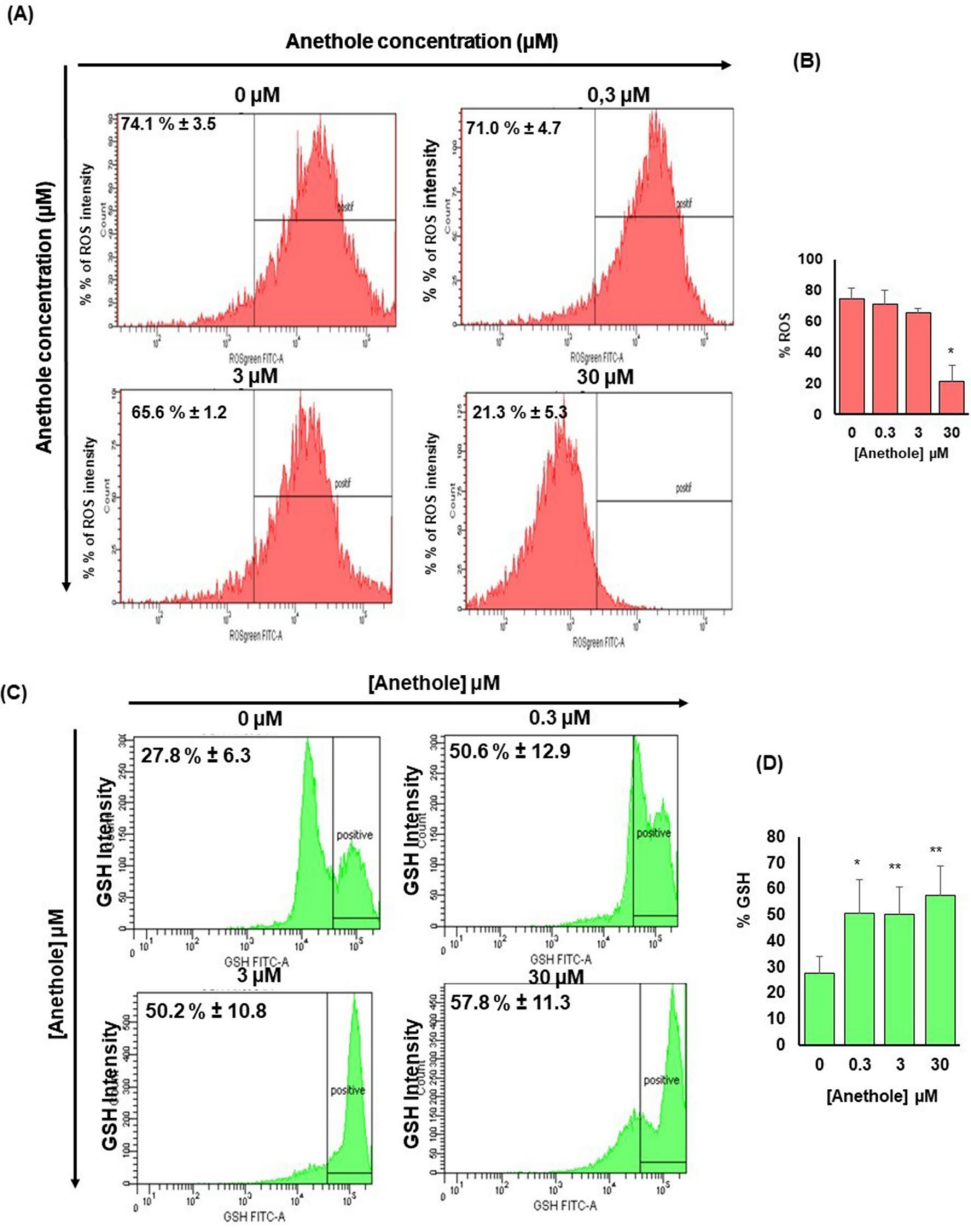
Anethole promotes the inhibition of oxidative stress in oral cancer cells. (**A**) The quantification of ROS by flow cytometry (n = 4) showed an inhibition of approximately 52% when the Ca9-22 cells were treated with anethole (30 μM) for 24 h. (**B**) D) Histogram showing fold changes of ROS between untreated cells and cells treated by anethole concentration. (**C**) The flow cytometry analysis of the intracellular GSH (n = 4) revealed an increase of approximately 29% after 24 h of stimulation with anethole. (**D**) Histogram showing fold changes of GSH expression. Results have been reported with means (%) ± SD. Significance was obtained when comparing the control to anethole treated cells, *p < 0.05.

We demonstrated that Ca9-22 cells treated with 30 μM is having a significant effect on ROS by reducing the level from the control (73.1% ± 3.0%) to 21.2% ± 5.3% (*p* = 0.04) for 30 μM of anethole (Fig. 5A,B). Anethole at 0.3 μM and 3 μM did not seem to have effect on ROS activity, since the percentages were respectively at 71.0% ± 4.7% (*p* = 0.2) and 65.6% ± 1.2% (*p* = 0.08). We also demonstrated that anethole increased the level of GSH, ranging from 27.8% ± 7.2% with no treated cells, to 50.6% ± 12.9% (*p* = 0.05) with 0.3 μM, to 50.2% ± 10.8% (*p* = 0.008) with 3 μM, and to 57.8% ± 11.3% (*p* = 0.005) with 30 μM (Fig. 5C, D). These are suggesting that anethole could reduce tumor development.

### Anethole exhibits potent anti-oral cancer properties through targeting several cancer-promoting pathways

**Figure 6:**
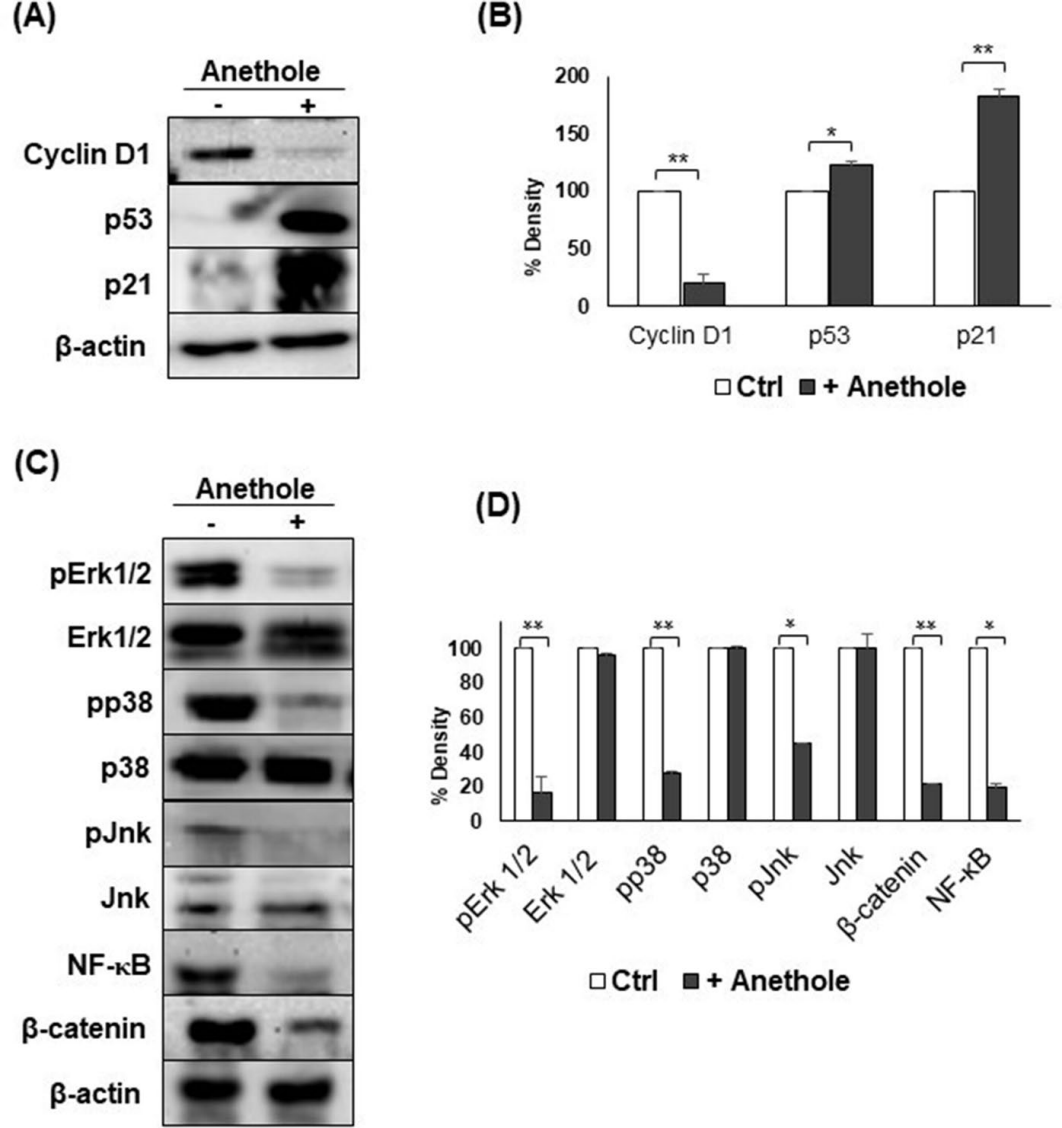
Anethole modulates the expression of several genes in oral cancer cells. Western blotting confirmed the involvement of anethole (10 μM) on mouth epidermoid carcinoma cells (Ca9-22) after being exposed for 24 h. Proteins were extracted with the Bradford technique before migration on SDS-PAGE (7 to 15%). (**A**) Anethole decreases oncogenic proteins and increases the expression of the tumor suppressor gene and the cell cycle inhibitor (n = 3). (**B**) Histogram displaying the three experiments of the cell cycle with western blotting reporting results as the means (% of density) ± SD. (**C**) Anethole inhibits the proliferative pathways MAPKinase (ERK 1/2, p38, and Jnk), Wnt (β-catenin) and NF-κB (n = 3). (**D**) Histogram showing the three experiments of signaling pathways with western blotting reporting results as the means (% of density) ± SD. Significance was obtained when comparing the control to anethole treated cells. *p < 0.05 or **p < 0.005.

In order to study the molecular pathway that regulates the induction of anti-oral cancer properties related to anethole, we investigated the effect of the latter on cancer-related pathways. As shown in Fig. 6, anethole has a strong inhibiting effect on the expression of cyclin D1. Furthermore, anethole increased the expressions of p53 and p21 (Fig. 6A,B). Anethole strongly inhibited the activities of MAPKases (ERK1/2, p38 and Jnk), NF-κB and Wnt pathway genes in Ca9-22 cells (Fig. 6C,D). These results indicate that anethole is an efficient inhibitor of several oral cancers related signaling pathways including MAPK/NF-κB and Wnt/β-catenin, leading to strong inhibition of cyclin D1. These data show that anethole is an efficient inhibitor for several cancer-promoting pathways.

### Anethole has anti-metastatic activity to reduce expressions of MMPs and to strongly enhance that of TIMPs in oral cancer cells

**Figure 7:**
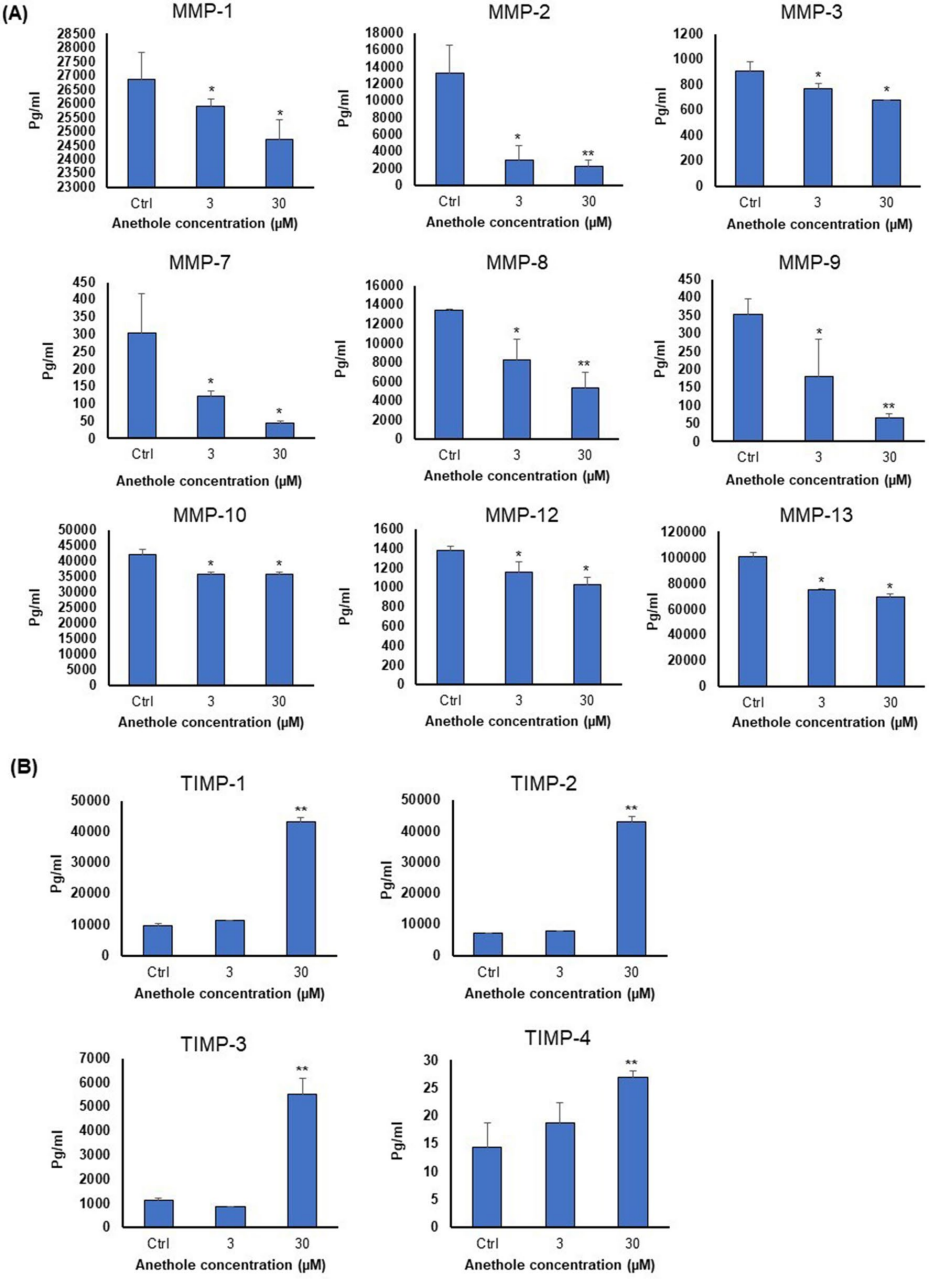
Anethole reduce the MMPs expression and increase TIMPs expression of oral cancer cells. Mouth epidermoid carcinoma cells (Ca9-22) were maintained in culture with or without anethole for 24 h. The Luminex xMAP technology for multiplexed quantification was used to measure the 9-plex of MMP and the 4-plex of TIMP. The analysis was performed using the Luminex 100 system (n = 3). (**A**) Anethole 3 μM and 30 μM significantly decreased the MMPs (− 1, − 2, − 3, − 7, − 8, − 9, − 10, − 12, and − 13) expression, compared to control. (**B**) Anethole 30 μM significantly increased TIMPs (− 1, − 2, − 3 and − 4) expression, compared to control. Significance was obtained when comparing the control to anethole treated cells. *p < 0.05 or **p < 0.005.

The fact that NF-κB controls the expression of several pro-metastatic cytokines such as MMPs and TIMPs prompted us to investigate anethole effect on matrix metalloproteinases (MMPs) and their inhibitor proteins (TIMPs) by Luminex xMAP technology. Figure 7A shows that anethole at 30 μM can reduce the level of MMPs by 85% inhibition for MMP-7 and 82% for MMP-9 in Ca9-22 cells, whereas for metalloproteinase inhibitors (TIMPs), it promotes expression, ranging from 1.09 to 6.09-fold higher (Fig. 7B). These data indicate that MMPs and TIMPs family is closely associated with oral tumorigenesis and may be an important potential prognostic marker for oral cancer progression.

## Discussion

In the current study, proliferation and apoptosis analysis provided evidence that anethole has anticancer properties in human oral squamous cell carcinoma, and could be considered as a potential therapeutic agent for oral cancer by disrupting their cell cycle, increasing cell apoptosis, and targeting MAPkases, NF-κB and Wnt pathways and the transcriptional activity of theses pathways. After treatment with 30 µM of anethole for 24 h, we are attending a high accumulation of cells in G2/M phase. The increase of the cells at the G2/M phase could be associated with apoptosis and with the level of DNA damage^22,23^. Blocking oral cancer cells at G2/M phase was strongly associated with the inhibition of Cdc2 dephosphorylation^24^ and to that of MAPKs known to induce the cell cycle arrest and apoptosis^25^. These results are consistent with previous studies reporting that anethole was considered as an interesting anti-cancer molecule for gastric cancer treatment by reduction of adenocarcinoma cells, triggering apoptosis and arresting cell cycle through mitochondrial pathways and the down-regulation of an oncogene, such as cyclin D1, known to be the downstream effector of the RAS/MEK/ERK pathway^12,26^. Cyclin D1 has been found highly expressed in cancer tumors and was associated with metastasis^27^. In addition, inhibition of MEK1/2 pathway by anethole significantly destabilized several oncoproteins, such as cyclin D1, p21, and β-catenin expressions. Inhibition of cyclin D1 is critical for destroying efficiently oral cancer cells. Wnt/β-catenin is considered as the important cancer pathway involved that has been found highly active in various types, including oral cancer^28,29^. In recent study by Eun Jeong Choo et al.^30^, it has been shown that anethole exerts anti-metastatic activity on human fibrosarcoma cells by suppressing phosphorylation of AKT, extracellular signal-regulated kinase (ERK), p38, and nuclear transcription factor-kappa B (NF-κB). Furthermore, anethole exhibits potent selective anti-oral cancer properties through targeting these cancer-promoting pathways in epithelial monolayers. Inhibition of these pathways can be an excellent target for cancer therapy. In addition, MAPK and Wnt pathways activated are a marker of poor prognosis in human oral cancer, and persistent MAPK activation in it is associated with enhanced cellular and tumor growth^31,32^. The high cytotoxic effect of anethole in oral cancer cells at low concentration but in normal gingival cells, it has a marginal effect at low concentration, but this molecule appears to have some toxicity when used at high concentration. Also, our results shown that anethole is cytotoxic and triggering apoptosis and autophagy as well as inducing oxidative stress. This data was confirmed by western blot analysis, which has clearly demonstrated that anethole induces apoptosis through activation of caspases 3 and 9 as well as PARP-1 cleavage. Anethole-induced apoptosis was controlled through a mitochondrial pathway via up-regulation of Bax/Bcl-2 ratio and with increased expression of regulatory genes in the cell cycle arrest as p53 and p21^WAF-1^ proteins. Same results were reported by Al-Sharif et al.^33^ in 2013 while using another phenolic natural product for breast cancer therapy. They indicated that eugenol exhibits anti-breast cancer properties by triggering apoptosis through the induction of the intrinsic pathway, by increasing p21^WAF1^ expression, by inhibiting cyclin D1 and ERK1/2, p38 and NF-κB signaling pathways that were known to play a key role in oral carcinogenesis. Our suggestion is that anethole suppresses the activation of MAPkase and induction of caspase cleavage; this is leading to the down-regulation of several MAPK targets, such as cyclin D1, p53, and caspases ones, as Bax, Bcl-2 and cytochrome C. All these proteins play key roles in oral tumorigenesis^34–36^.

Second, we demonstrated that anethole exhibits anti-carcinogenic activities through another physiological phenomenon called autophagy, as a target for treatments of oral cancer diseases. Indeed, flow cytometry analysis indicated the activation of autophagy, an important cell death process, in oral cancer cells, in presence of anethole. In Ca9-22 cells, the basic level of autophagy seems to be very low and while treated with anethole, we assist to p53 suppression causing a significant increase of the phenomenon and leading to cell death. In this order, several previous studies have reported that autophagy was discovered in inducing G0/G1 arrest and apoptosis of cancer cells via β-catenin pathway^37^. We can suggest that autophagy may be related to the apoptosis induction by anethole treatment of oral cancer cells by increasing the Bax/Bcl-2 ratio and activating the subsequent caspases. The anti-oral cancer properties of this treatment was associated with the reduction of oxidative stress, also an important factor leading to the formation and progression of cancer^38^. Although, anethole has been known as an antioxidant and an inhibitor of lipid peroxidation, as well as having an important role in regulating activities of mTOR/PPARγ and ROS control^39^. These functions of anethole, as an antioxidant, depend on how ROS are efficiently regulated both inside and outside the cell. The facts that anethole has the ability to modulate positively, by decreasing the presence of reactive oxygen species, is another indicator of its anticancer potential. In addition, these results correlate with an increase in intracellular GSH, a mediator that plays a crucial role in cellular processes such as differentiation, proliferation and apoptosis, and indicate an inhibition of cancer progression^40^. Anethole was reported playing a key role as a protective agent against radiation- and ROS-induced cytotoxicity, also known to be an activator of the enzyme gamma-glutamylcysteine synthetase, which is involved in GSH synthesis^39,41^. It is thus able to maintain the redox status of ROS-stimulated cells by increasing the intracellular level of GSH^42^.

The metastases are directly associated with induction of EMT destruction, MAPK and Wnt pathways^43,44^. In the current study, we have demonstrated that anethole inhibits the epithelial-to-mesenchymal transition in oral cancer through blocking migration/invasion abilities of cells and suppressing the EMT via the enhancement of the epithelial marker E-cadherin, attenuating the prominent mesenchymal marker (vimentin) and reducing the MMPs secretion. It previously reported that anethole strongly inhibits the migration of prostate cancer cells by regulating the epithelial-to-mesenchymal transition and MMPs, which are considered, with their tissue inhibitors, as key factors for metastatic tumors^45^. There is accumulating evidence that MMP-9 potentiates various cancer metastasis^46,47^. It has a powerful role to destroy collagen IV and other ECM components^48^. It clearly reported by scientists that MMP-9 is a strong candidate to be a marker for different types of cancer^49,50^. TIMPs inhibitors of MMPs were also indicated to be associated with the same cancer^51^. Experimental reports demonstrated direct implication of TIMPs in the majority of hallmarks and showed their deregulation in most of the tumors^51^.

## Conclusion

**Figure 8:**
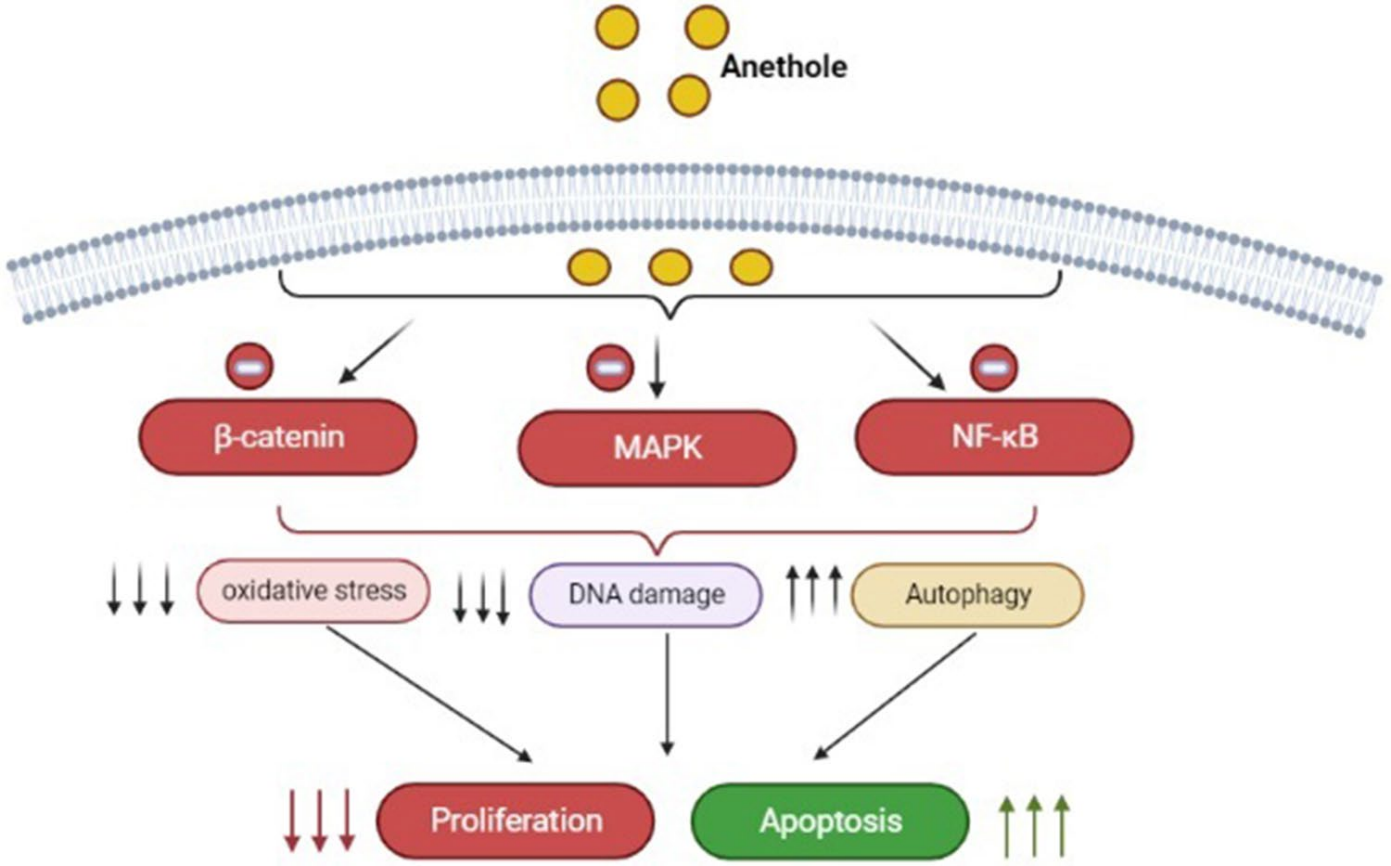
Schematic diagram showing the hypothesized mechanism of anethole-induced selective killing of human oral cancer Ca9-22 cells. In Ca9-22 treated with anethole, enhanced activation of apoptosis pathways against the inhibition of proliferative pathways.

Our results confirm that the anethole treatment selectively induces anti-oral cancer proprieties via shifting proliferative signaling pathways and by triggering apoptosis, autophagy and oxidative stress (Fig. 8). Anethole shows potential as a natural product possessing potent therapeutic activity and show very promising effects as regards to its use in the treatment of gum cancer. Further studies in animals and humans will help in translating these results in vivo.

## Supplementary Information

Supplementary Information.

## Abbreviations

AKT: PKB: Protein kinase B
Bax: Bcl-2-associated X protein
Bcl-2: B-cell lymphoma 2
BRAF: Serine/threonine-protein kinase B-raf
Ca9-22: Cancer gingival cells
DME: Dulbecco’s modified Eagle’s
DMEH: Dulbecco’s modified Eagle’s/Ham’s
DMSO: Dimethyl sulfoxide
OD: Optical density
ECM: Extracellular matrix
EDTA: Ethylenediamine tetraacetic acid
EGFR: Epidermal growth factor receptor
EMT: Epithelial-to-mesenchymal transition
ERK1/2: Extracellular signal-regulated kinases
FBS: Fetal bovin serum
GEC: Gingival epithelial cells
GF: Gingival normal fibroblasts
GSH: Gluathione
Jnk: C-Jun N-terminal kinases
KRAS: Ki-ras2 Kirsten rat sarcoma viral oncogene homolog
LDH: Lactate deshydrogenase
MAPK: Mitogen-activated protein kinases
MEK: Rapidly accelerated fibrosarcoma
mTOR: Mechanistic target of rapamycin
MMP: Matrix metalloproteinases
MTT: 3-(4,5-Dimethylthiazol-2-yl)-2,5-diphenyl tetrazolium bromide
NF-κB: Nuclear factor-kappa B
p21: Cyclin-dependent kinase inhibitor 1
p38: P38 mitogen-activated protein kinase
p53: Tumor protein 53
P62: Ubiquitin-binding protein p62/Sequestosome-1
PARP1: Poly [ADP-ribose] polymerase 1
PBS: Phosphate-buffered saline
Pi: Propidium Iodide
PPARγ: Peroxisome proliferator-activated receptor gamma
PVDF: Polyvinylidene difluoride membrane
ROS: Reactive oxygen species
RPMI: Roswell Park Memorial Institute medium
SDS-PAGE: Sodium dodecyl sulfate polyacrylamide gel electrophoresis
TBS: Tris-buffered saline
TIMP: Metalloproteinase inhibitors
Wnt: Wnt signaling pathways
