# Supplementary material for: Anethole induces anti-oral cancer activity by triggering apoptosis, autophagy and oxidative stress and by modulation of multiple signaling pathways
Source: Sci Rep. 2021 Jun 22;11:13087. doi: 10.1038/s41598-021-92456-w (PMC8219795; doi:10.1038/s41598-021-92456-w)
Supplement: Supplementary file 1 — Supplementary Information. [file 41598_2021_92456_MOESM1_ESM.pptx]

## Slide 1
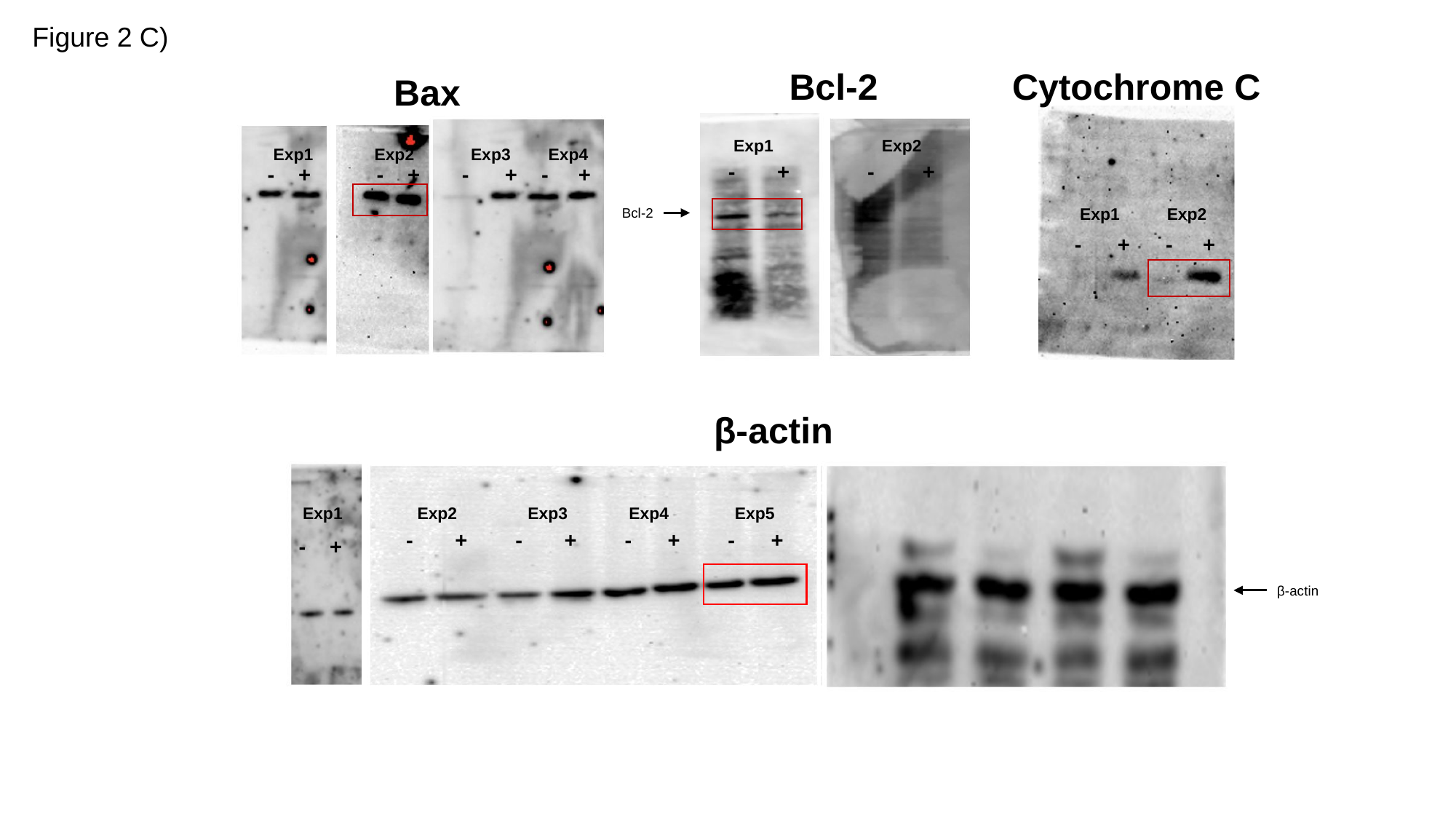

Figure 2 C)
Bcl-2
Cytochrome C
Bax
Exp1 Exp2
Exp1 Exp2 Exp3 Exp4
- + - +
- + - + - + - +
Exp1 Exp2
Bcl-2
- + - +
β-actin
Exp1
Exp2 Exp3 Exp4 Exp5
- + - + - + - +
- +
β-actin

## Slide 2
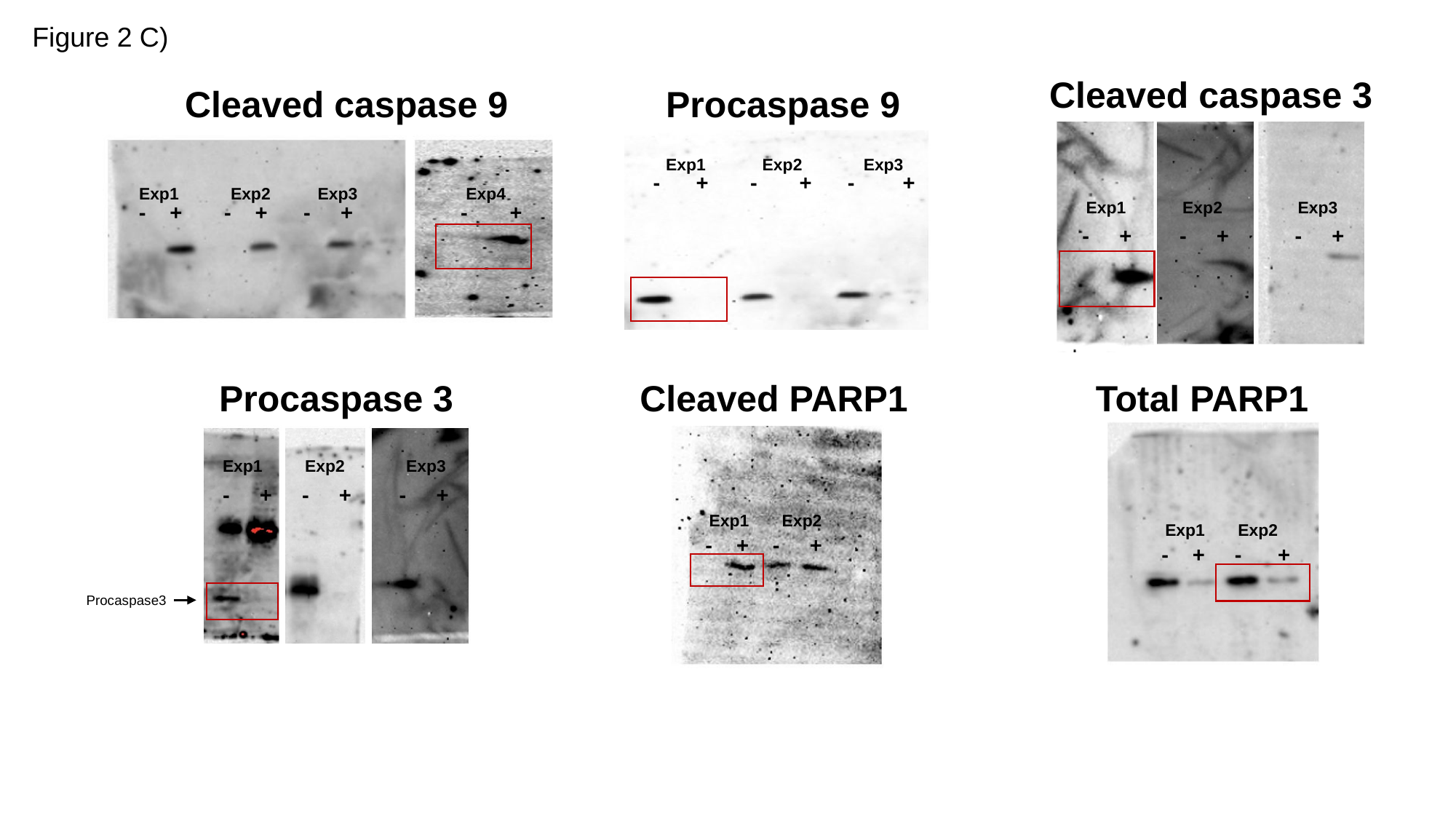

Figure 2 C)
Cleaved caspase 3
Cleaved caspase 9
Procaspase 9
Exp1 Exp2 Exp3
- + - + - +
Exp1 Exp2 Exp3 Exp4
Exp1 Exp2 Exp3
- + - + - + - +
- + - + - +
Total PARP1
Procaspase 3
Cleaved PARP1
Exp1 Exp2 Exp3
- + - + - +
Exp1 Exp2
Exp1 Exp2
- + - +
- + - +
Procaspase3

## Slide 3
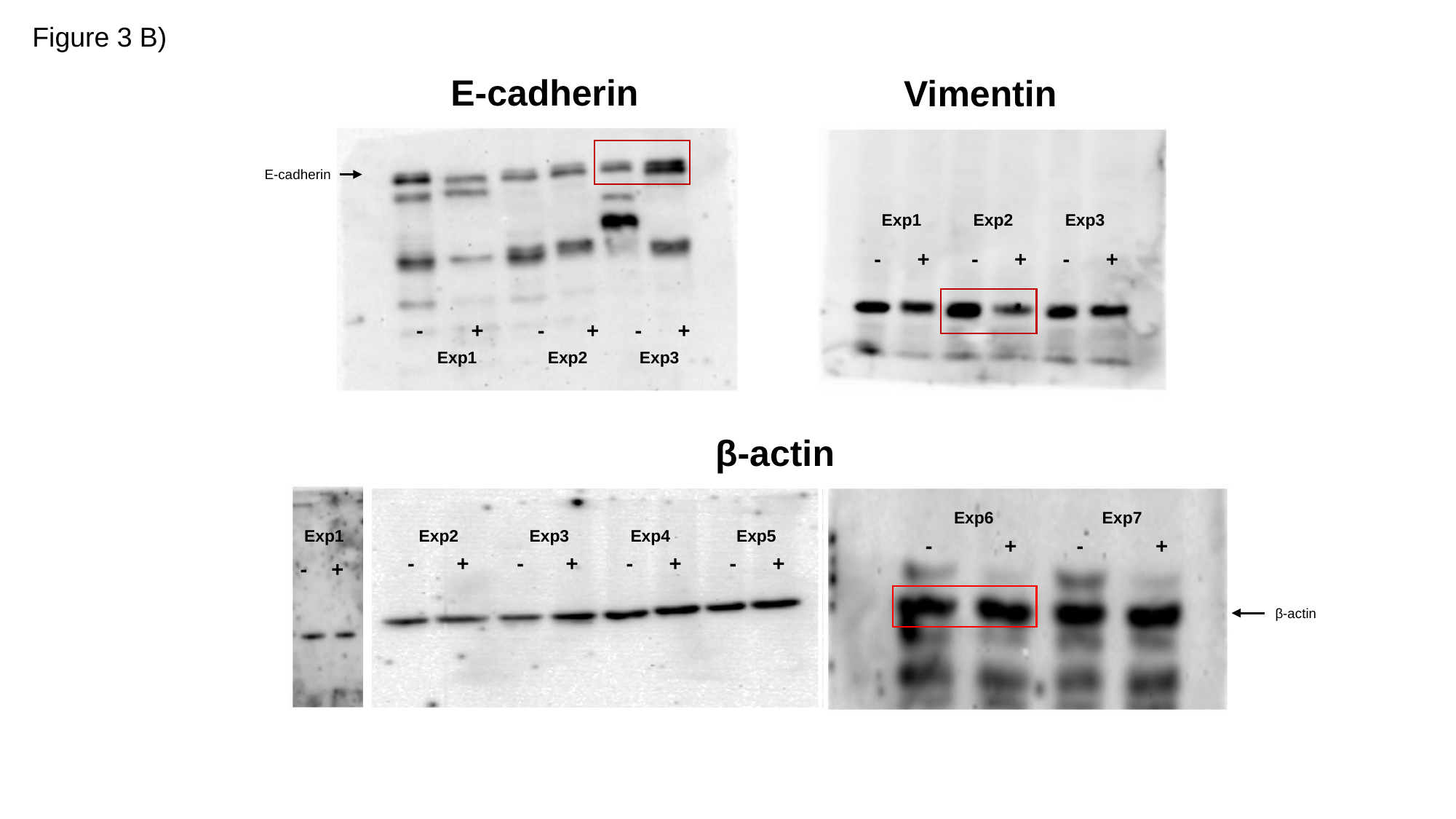

Figure 3 B)
E-cadherin
Vimentin
E-cadherin
Exp1 Exp2 Exp3
- + - + - +
- + - + - +
Exp1 Exp2 Exp3
β-actin
Exp6 Exp7
Exp1
Exp2 Exp3 Exp4 Exp5
- + - +
- + - + - + - +
- +
β-actin

## Slide 4
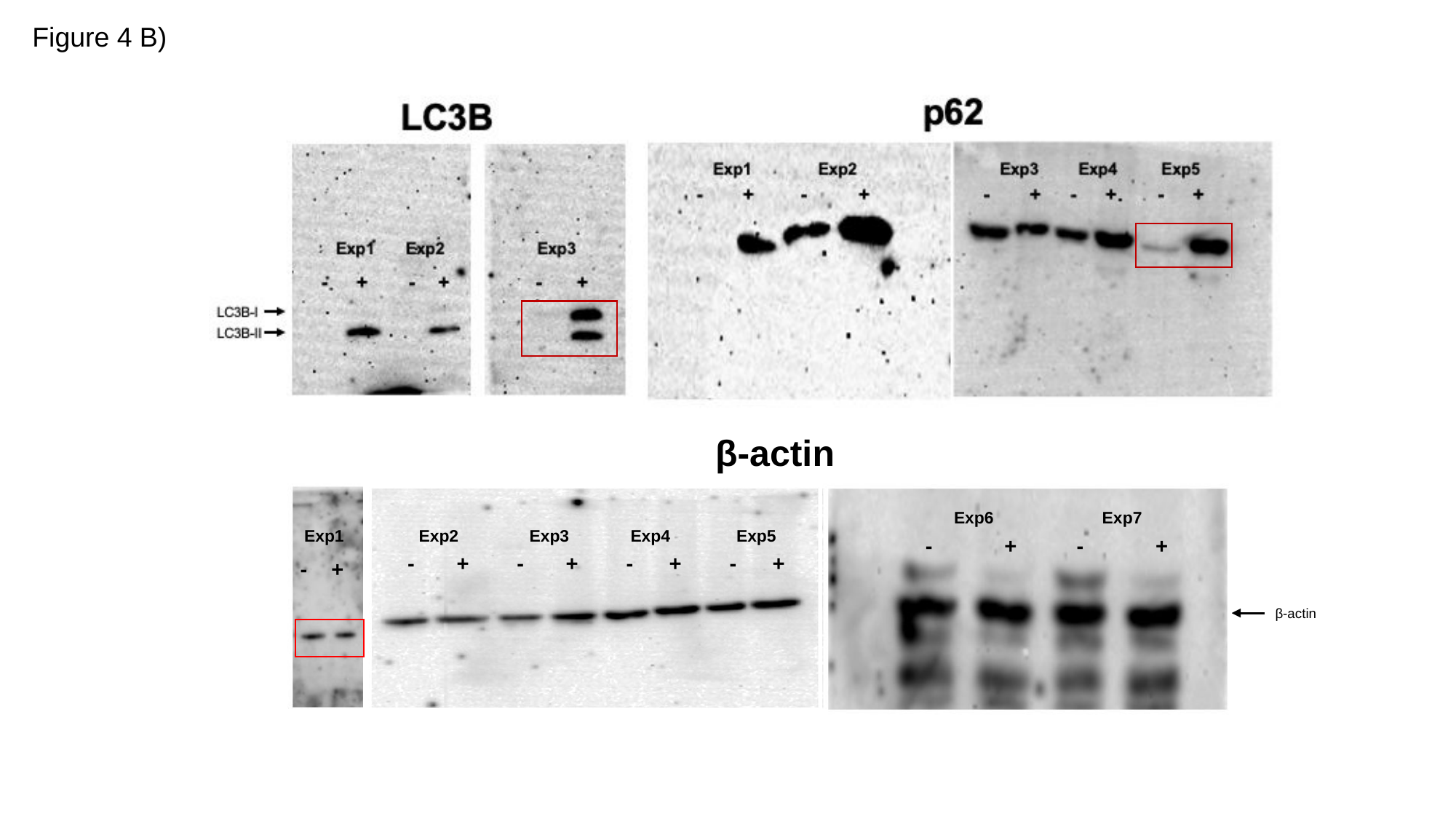

Figure 4 B)
β-actin
Exp6 Exp7
Exp1
Exp2 Exp3 Exp4 Exp5
- + - +
- + - + - + - +
- +
β-actin

## Slide 5
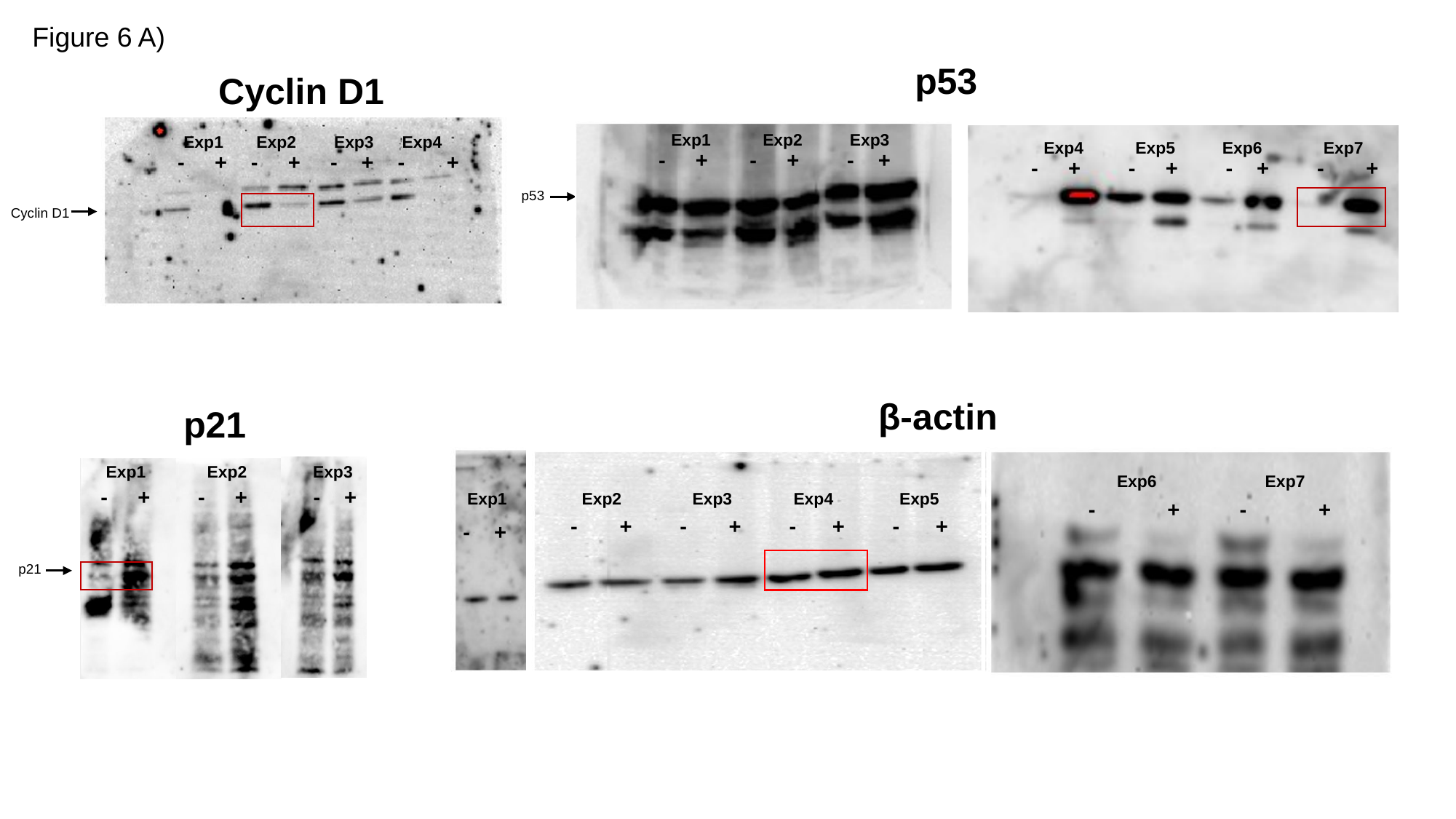

Figure 6 A)
p53
Cyclin D1
Exp1 Exp2 Exp3
Exp1 Exp2 Exp3 Exp4
Exp4 Exp5 Exp6 Exp7
- + - + - +
- + - + - + - +
- + - + - + - +
p53
Cyclin D1
β-actin
p21
Exp1 Exp2 Exp3
Exp6 Exp7
- + - + - +
Exp1
Exp2 Exp3 Exp4 Exp5
- + - +
- + - + - + - +
- +
p21

## Slide 6
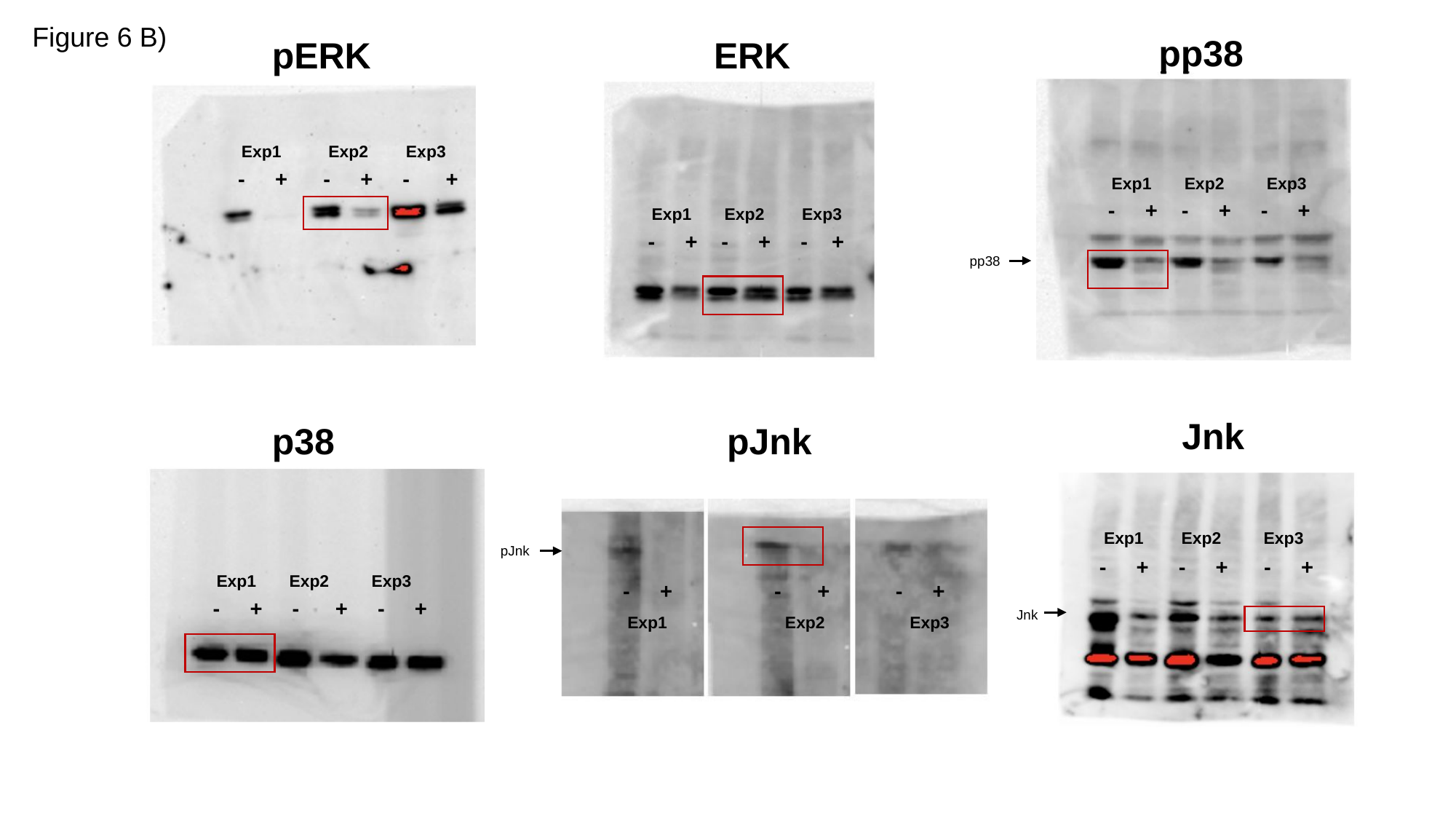

Figure 6 B)
pp38
pERK
ERK
Exp1 Exp2 Exp3
- + - + - +
Exp1 Exp2 Exp3
- + - + - +
Exp1 Exp2 Exp3
- + - + - +
pp38
Jnk
pJnk
p38
Exp1 Exp2 Exp3
pJnk
- + - + - +
Exp1 Exp2 Exp3
- + - + - +
- + - + - +
Jnk
Exp1 Exp2 Exp3

## Slide 7
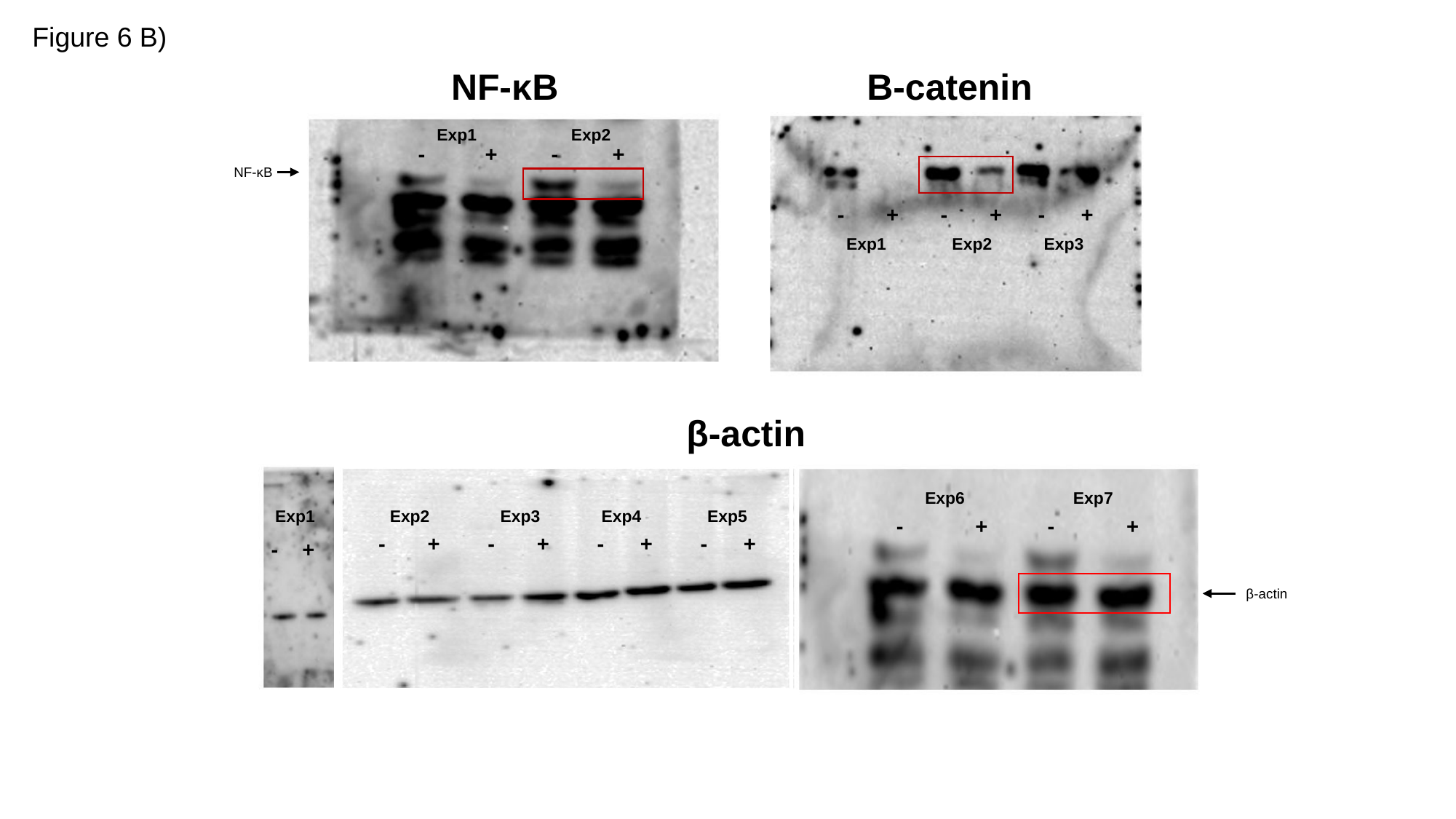

Figure 6 B)
Β-catenin
NF-κB
Exp1 Exp2
- + - +
NF-κB
- + - + - +
Exp1 Exp2 Exp3
β-actin
Exp6 Exp7
Exp1
Exp2 Exp3 Exp4 Exp5
- + - +
- + - + - + - +
- +
β-actin
